# Effect of Chamomile Hydroalcoholic Extract on Bleomycin-Induced Pulmonary Fibrosis in Rat

**Published:** 2018-10

**Authors:** Ali Asghar Hemmati, Amir Jalali, Parastoo Keshavarz

**Affiliations:** 1 Department of Pharmacology, School of Pharmacy and Medicinal Plants Research Center, Ahvaz Jundishapur University of Medical Sciences, Ahvaz, Iran,; 2 Department of Toxicology, School of Pharmacy and Toxicology Research Center, Ahvaz Jundishapur University of Medical Sciences, Ahvaz, Iran,; 3 Department of Pharmacology and Toxicology, School of Pharmacy, Guilan University of Medical Sciences, Rasht, Iran

**Keywords:** Pulmonary fibrosis, Bleomycin, Chamomile hydroalcoholic extract

## Abstract

**Background:**

The aim of the present study was to investigate the effect of the Chamomile hydroalcoholic extract on bleomycin-induced pulmonary fibrosis in rat.

**Materials and Methods:**

Rats (N.Mari, 180–220 g) of either sex were given a single intratracheal instillation of bleomycin (7.5 IU/Kg) or the vehicle (saline). Treatment groups were given the same dose of bleomycin and then received different doses of oral chamomile hydroalcoholic extract (400, 600, 800, 1000, and 1500 mg/kg/day) for two weeks.

**Results:**

Histological and pharmacological experiments of bleomycin-treated animals showed that bleomycin could cause marked pulmonary fibrosis within two weeks. In addition, administration of Chamomile hydroalcoholic extract reduced such damages in lung tissue in a dose-dependent manner. Best results were obtained with 1500 /kg/day of Chamomile hydroalcoholic extract.

**Conclusion:**

From the results of current study, it can be concluded that Chamomile hydroalcoholic extract may be able to diminish the toxic effects of bleomycin on the lung tissues. Such effect of Chamomile can be attributed to the ingredients of this plant with anti-inflammatory and anti-oxidant properties.

## INTRODUCTION

Bleomycin is an antibiotic reagent with anti-tumor, anti-viral and antibacterial properties. Bleomycin is generally used in multi-drug chemotherapy due to its minimum bone marrow and immune system depression and is considered as a typical cytotoxic anti-neoplastic agent (1). Nonetheless, administration of Bleomycin may result in severe and chronic pulmonary fibrosis. DNA conjugation and production of superoxide apart from other oxygen activated reagents and hydroxyl radicals, are reported as bleomycin mechanisms of action (2, 3). It is assumed that this abnormality is a marker of inflammatory response in the alveolus. In one type of alveolus damage interstitial edema and accumulation of inflammatory cells occur (alveolitis) (4). Although bleomycin-induced damage to endothelial cells and subsequent migration of leukocytes ruins pulmonary protective barriers, there are many parameters such as chemotactic factors proliferation, synthesis and release of cytokines and leukotrienes and other pulmonary inflammatory mediators which advance damages (5).

Previous studies demonstrate the efficacy of herbal drugs in inflammation treatment. *Matricaria chamomilla* L. (*M. chamomilla*), from Compositea family has protective and anti-inflammatory properties and different pharmacological activities such as antigenotoxic effect (6), prevention of diabetic complication (7), anti-neoplastic effect on breast and uterine cancer cells in vitro (10–100 μg/ml concentration) (8), benzodiazepine-like activity (9) and phosphodiestrase inhibitory action (leads to increased cAMP levels) (10).

Although few treatment methods have been suggested to prevent the primary and advanced state of pulmonary fibrosis (5), there is no reliable treatment for pulmonary fibrosis. Despite many factors causing pulmonary disease, clinical symptoms and manifestations are to some extent similar (4, 5). Therefore, many fibrotic models induced by bleomycin were suggested in order to prevent primary and advanced pulmonary fibrosis. In addition, since inflammation has a major role in pulmonary fibrosis occurrence, administration of steroid and non-steroidal anti-inflammatory agents were evaluated. Apart from that corticosteroids (11) drugs such as indomethacin and sodium diclofenac can decrease pulmonary collagen accumulation (4–7). It should be noted that administration of these drugs with efficient dose for prevention of collagen accumulation might cause non-pulmonary toxicity (12).

Herbal parts containing 0.2–0.8% essence are widely used. The Chamomile essence contains Camazolene as an anti-inflammatory agent, sesquiterpene type B and C, Camomilol, caproic and nonoleic ethers, apigenin, salicylic acid and flavonids such as rutin, valeric acid and tannins (11–13).

The aim of the present study was to evaluate the anti-inflammatory and anti-oxidant effects of *M. chamomilla* plant in bleomycin-induced pulmonary fibrosis.

## MATERIALS AND METHODS

Bleomycin sulfate was purchased from Nippon Kayaku Co. (Tokyo, Japan). Ketamine hydrochloride was received from Gedeon Richter, Hungary. Sodium thiopental was gifted by Rhone Poulenc Rorer, France. Sodium tungsten was purchased from BDH, England. Other reagents were received from Merck Co, Germany. All other reagents were highly commercially available. Chamomile dried flowers also were obtained from *M. chamomilla* plant, *Compositae* family, GolDaru company, Isfahan, Iran.

### Preparation of the hydroalcoholic *M. chamomilla* extract

Dried flowers of *M. chamomilla* were obtained at late autumn from GolDaru Co. Isfahan, Iran. The dried flowers were taxonomically identified at the Department of Botany, School of Sciences, Ahvaz University, Ahvaz, Iran. The dried flowers were cut into small pieces, crushed, saturated with 70% ethanol and 30% water, being macerated at room temperature (25±3 °C) for 5 days. The suspension was filtered through muslin cloth and the filtrate was collected. The residual of *M. chamomilla* powder was re-extracted with ethanol-water for three times. The supernatant was rotary evaporated to dryness, using vacuum evaporator (Adolphe, Model 462, Germany). This hydroalcoholic extract was kept in the refrigerator for all experiments. Then, certain volumes for each dose preparation were calculated.

### Animals

Experiments were performed on N.MARI rats of either sex (180–220 g) which were purchased from the Razi Hesarak Institute, Karaj, Iran. Animals were housed in animal house of Jundishapur University of Medical Sciences, Ahvaz, Iran. Animals were fed on a standard, commercial, pellet diet (Shushtar Khorakdam Co., Iran). In addition, they were supplemented with fresh vegetables and tap water. The animal house temperature was adjusted at 24±2 °C and a 12:12 hour light: dark cycle was maintained while the study was carried out.

The Ethic Committee of the Jundishapur University, Ahvaz approved the design of the experiments, and the protocol confirms to the guidelines of the National Institutes of Health (NIH).

### Experimental groups and treatment regimens

Experimental animals were divided into three major groups.

Group-I. Controlled animal group received intratracheal normal saline (n=5).
Group-II. Animals received intratracheal bleomycin sulfate (n=5).
Group-III. Animals received *M. chamomilla* hydroalcoholic extract by oral gavages (400, 600, 800, 1000, 1500 mg/kg) for a period of two week after bleomycin sulfate intratracheal administration (n=5 for each dose).

The animals were anesthetized by thiopental (120 mg/kg). The control group received 0.2 ml normal saline, while groups II and III received simultaneous intratracheal administration of 0.2 ml solution of bleomycin 7.5 U/ml dissolved in normal saline.

### Histological evaluation

After a two weeks period the experimental animals were anesthetized with 0.3 ml thiopental (120 mg/kg) and scarified and then lungs were removed; the left lobe of lung tissue was fixed in 10% formalin solution for histological studies. In the next step a mid- sagittal section of each lung was prepared at 5 μm thickness and stained with Hematoxylin and Eosin (H&E). In order to evaluate the pathological grade caused by inflammation and fibrosis in the whole area and connective tissue changes apart from interstitial fluid particularly collagen, the prepared samples were stained with reticulin. In the following, the sections were evaluated by light microscopy.

### Pharmacological experiments

In the recent study, isolated lung tissues were used for i*n-vivo* pulmonary contractility study. After death, both heart and lungs were removed and maintained in Krebs solution. A mid-sagittal section was prepared from lung tissue with 5×5×20 mm and 50–80 mg weight. The lung tissue was maintained in the isolated organ bath at 37 °C with permanent perfusion of 95% oxygen and 5% CO _2_ . The prepared section was attached to an isometric transducer (Harvard, UF1). 1 g-tension was induced in relaxation state for 45 min. The tissue contractions were recorded by a Harvard student polygraph. Each 2 cm vertical movement of pen was calibrated for 500 mg tension concerning with tissue contraction. Contraction reagents were depolarization potassium solution containing 4.475, 8.95, 17.9 mg *K*^+^ and sodium tungsten 10^−3^ –10^−8^ M bath concentration.

### Statistical analysis

After addition of contraction constituents to bath and obtaining pulmonary contraction responses, the tension (mg/mg weight) against concentration was plotted. In this study, the results were expressed as mean ± SD. Paired Student’s t-test and analysis of variance were performed in order to determine the significant differences between means. P< 0.05 was considered statistically significant.

## RESULTS

### Histological results

**Figure 1.**
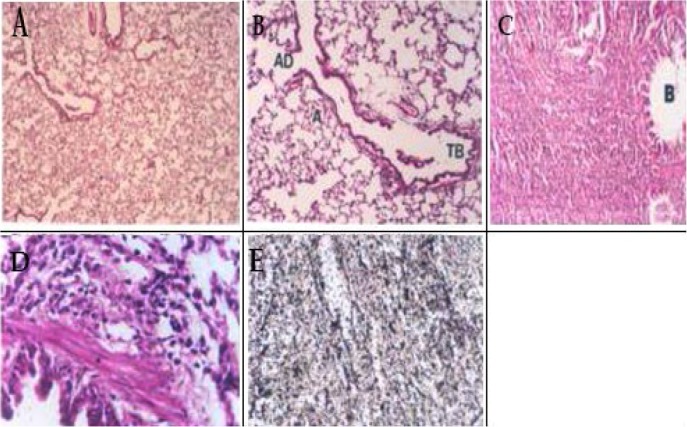
Light microscopic figures of lung section from rat following 2 weeks intratracheal administration. Hematoxylin and Eosin (H&E). (**A**) given normal saline (NS): alveolus completely open with thin alveolar wall without any inflammation or fibrosis (H&E×40); (**B**) given normal saline: terminal bronchiole (TB), alveolar duct (AD) and alveoli are seen well (H&E×100); (**C**) given bleomycin: inflammatory cells accumulation including lymphocytes, fibroblasts and myofibroblasts; interstitial tissue thickness is seen (B=bronchiole) (H&E×200); (**D**) Bronchiole smooth muscle (▲) inflammatory cells, fibroblast and myofibroblast (↑)(H&E×200); (**E**) collagen sedimentation as black color (Reticulin ×100).

**Figure 2.**
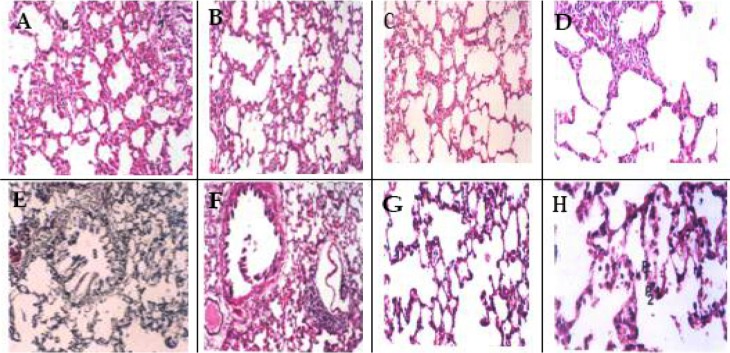
Light microscopic figures of lung section from rats following 2 weeks administration (**A**) Administration of the *Matricaria chamomilla* extract 400 mg/kg: alveolar space is accumulated by inflammatory cells (A), thickening and collagen sedimentation (C) (H&E×100); (**B**) Administration of the *Matricaria chamomilla* extract 600 mg/kg: reduction in tissue thickness along with inflammatory cells are seen (H&E×100); (**C**) Administration of the *Matricaria chamomilla* extract 800 mg/kg; expansion the alveolar space in some areas and in some areas accumulation of inflammatory cells are seen (H&E×100); (**D**) Administration of the *Matricaria chamomilla* extract 800 mg/kg: Inflammatory cells are well seen (H&E×200); (**E**) Administration of the *Matricaria chamomilla* extract 800 mg/kg: collagen sedimentation is decreased dramatically around bronchiole (B) and alveolar space (Reticulin ×100); (**F**) Administration of the *Matricaria chamomilla* extract 1000 mg/kg: reduction in alveolar wall thickness and inflammatory cell infiltration are seen (H&E×100); (**G**) Administration of the *Matricaria chamomilla* extract 1500 mg/kg: alveoli completely open, alveolar thickness in normal status (H&E×200); (H) Administration of the *Matricaria chamomilla* extract 1500 mg/kg: pneumocytes I (P1) and II (P2) are seen (H&E×400).

After a two-week period of bleomycin intratracheal administration, pathological changes and fibrotic damages were evaluated by comparing prepared microscopic slides with control group and group receiving Chamomile hydroalcoholic extract. The results indicate that no hemorrhagic damage was observed in the control group that received normal saline, alveolus were completely open, with thin alveolar wall and thin interstitial fluid. In the group that received bleomycin sulfate, inflammatory cells accumulation, including lymphocytes, and plasma cells were observed. In addition myofibroblasts and fibroblasts proliferations, alveolar tissue collapse, interstitial fluid thickening following collagen accumulation and finally fibrosis were observed. Pulmonary injuries were distributed in whole pulmonary lobes (Figure 1). In the group that received Chamomile hydroalcoholic extract (400 mg/kg) after two weeks intratracheal bleomycin sulfate administration, the inflammatory cells occupied alveolar spaces and collagen sedimentation was elucidated in alveolar spaces. In the group that received 600 mg/kg Chamomile hydroalcoholic extract, the thickness of interstitial tissues was decreased, although inflammatory cells were dramatically increased. On the other hand, in the group that received 800 mg/kg Chamomile hydroalcoholic extract the alveolar space was expanded in some areas and in some areas accumulation of inflammatory cells and fibrosis were observed. In the group that received Chamomile hydroalcoholic extract (1000 mg/kg) although alveolar wall thickness was reduced, inflammatory cell infiltration was observed in some areas. In the group that received Chamomile hydroalcoholic extract (1500 mg/kg), alveoli were completely open, and alveolar thickness was in the normal status (Figure 2). Microscopic tissue studies illustrate that in experimental animals that received Chamomile hydroalcoholic extract, intensity of pulmonary fibrosis was less than the group that received just bleomycin sulfate in a dose-dependent manner. In addition, the highest efficacy was observed when 1500 mg/kg Chamomile hydroalcoholic extract was administered ( Figures 1 and 2).

### Pharmacological results

**Figure 3.**
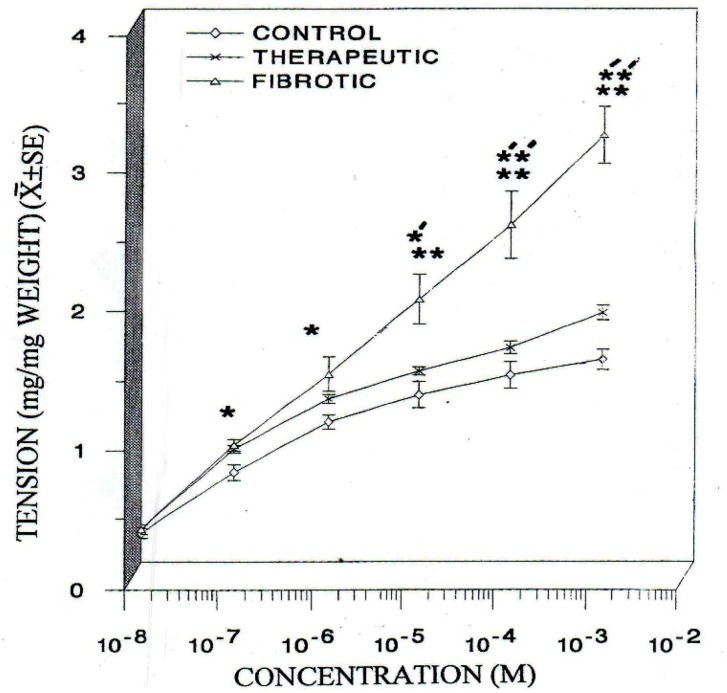
The comparison of lung strip contractility between control (given normal saline), given 1500 mg/kg extract, and Fibrotic groups *: P<0.05 significant made with control group, **: P<0.01 significant made with control group, *” :P<0.05 significant made with given 1500 mg/kg extract group, **”: P<0.01 significant made with given 1500 mg/kg extract group

In the recent study, lung tissue in group receiving 1500 mg/kg Chamomile hydroalcoholic extract was studied in order to determine contractility. Control and fibrotic groups were compared for more pharmaco-mechanic evaluation. The left lobe was selected for study because it has few bronchial smooth muscular cells. Fibrotic tissues that received both sodium tungsten and potassium depolarized solution showed more contractility in contrast to the control group. Sodium tungsten-induced contractions in normal group, while treated group with Chamomile extract showed no significant difference in concentration range of 10^−4^–10^−8^ M, however, at 10^−3^ M contractility in the treated group was more in comparison to control group (P<0.05). In addition, no significant difference was observed concerning with fibrotic and treated group at concentration of 10^−6^–10^−8^ M sodium tungsten. The fibrotic group demonstrated more response in contrast to treated group with dose enhancement (Figure 3). The results illustrate that the contraction response in treated group was more in comparison to control group at 17.9 mg *K*+ and showed no significant difference in lesser amounts. In addition, a significant difference was observed between fibrotic and treated groups at contents of 4.47, 8.95 and 17.9 mg *K*+ solution, respectively (P<0.05, 0.01) (Figure 3).

## DISCUSSION

Pulmonary fibrosis is a dangerous disease which changes lungs normal structure and could cause threatening consequences in patients. Since pulmonary fibrosis is considered an inflammatory response, it can affect endothelial cells, fibroblasts and other pulmonary cells and cause advanced tissue damages through continuous inflammatory reactions. The most important manifestations are fibroblasts and myofibroblasts enhancement as well as collagen production and its accumulation in the alveolar spaces (12). Many inflammatory mediators such as prostaglandins, leukotrienes, free radicals and cytokines e.g. Platelet-Derived Growth Factor (PDGF), Fibroblast Growth Factor (FGF), and Tumor Necrosis Factor (TNF) are getting involved in fibrosis procedure (13).

The anti-inflammatory potential of *Chamomilla recutita* was shown clinically in patients with phlebitis due to antineoplastic chemotherapy (6). Chamomile caused significant inhibition on proinflammatory cytokines production IL1β, IL-6 and TNF-α (14). It was shown that Chamomile is a selective COX-2 inhibitor with anti-inflammatory activities (15). All these data showed that Chamomile has well-known anti-inflammatory and antioxidant activities. It was also demonstrated that anti-inflammatory effects may be mediated through inhibition of Nitric Oxide (NO) production (14).

Different studies were performed in regard to prevention and treatment of pulmonary fibrosis by administration of vitamin E (5) or non-steroidal anti-inflammatory drugs such as indomethacin and diclofenac (13). Since Chamomile plant contains great amounts of anti-inflammatory agents it can also be used to prevent and treat pulmonary fibrosis (11).

In the recent studies, the effect of the Chamomile hydroalcoholic extract on pulmonary fibrosis was evaluated. They demonstrated that herbal flavonoids can inhibit the production of prostaglandins and leukotrienes in rat (14, 15). Histological evaluation illustrates that the Chamomile hydroalcoholic extract can remarkably decrease pulmonary fibrosis damages which confirms previous studies.

Among flavonoid chamomile components, quercetin, apigenin, 7-O-glucoside and rutin are especially considered. Quercetin has been associated with antioxidant activities in *in vitro* model (16). Apigenin and its 7-O-glucoside derivative have prominent spasmolytic and antiphlogistic effects (17). Other biologically active compounds, including essential oils and terpenoids, bisabolol and chamuzulene have been shown to possess anti-allergic, antibacterial, antipyretic, ulcer-protective and antifungal properties (18). As shown apigenin along with its derivative as apigenin-7-O-glucoside are major bioactive compound in Chamomile extract aqueous and methanolic extract (19). Since anti-fibrogenic activity was observed with hydroalcoholic extract, these results signify apigenin to be considered as the major bioactive compound with anti-fibrogenic activity.

Pharmacological evaluations were also performed in order to prove that in normal lung tissue, prepared from left lobe, a limited number of smooth muscular cells and myofibroblasts exist; whereas in the fibrotic tissue, although the number of smooth muscle cells are approximately constant, myofibroblasts dramatically enhance for the aim of fibrosis induction and collagen production; therefore, specific myofibroblast-stimulating factors such as sodium tungsten can be used in order to determine fibrosis intensity. In addition, depolarized potassium solution also affects smooth muscle and myofibroblasts and contracts fibrotic tissue. The results demonstrate that fibrotic contraction capacity enhancement is due to the increase in myofibroblasts and fibroblast number which is confirmed by histological evaluation. Less contractility at higher doses of the Chamomile hydroalcoholic extract was observed due to the inhibitory effect of extract on inflammatory mediators release, decrease in myofibroblasts and fibroblasts activity which consequently decrease collagen synthesis and fibrosis. Although we have no ability to predict Chamomile extract mechanisms in the treatment of pulmonary fibrosis for certain, the antioxidant and anti-inflammatory of Chamomile extract particularly due to presence of Chamazulene is evident and it inhibits collagen, leukotrienes and prostaglandins synthesis.

A part from medicinal properties, several reports has raised concern about the toxic effects of Chamomile such as allergic reactions following oral administration (20) and contact dermatitis following topical applications (21, 22). Furthermore, Chamomile preparations contain components, especially chamazulene, Cis-spiroether and trans spiroether which showed in vitro inhibitory activities of major human drug metabolizing enzymes (23). Thus, the potential of interaction between Chamomile and drugs should be considered.

In the present study the anti-inflammatory potential of *Chamomilla recutita* was shown. As a conclusion, administration of Chamomile extract due to its advantages is suggested in patients receiving long-term bleomycin sulfate or subjects exposed to fibrogenic materials or fibrotic damages.

## References

[1] Tunon H Olavsdotter C Bohlin L Evaluation of anti-inflammatory activity of some Swedish medicinal plants. Inhibition of prostaglandin biosynthesis and PAF-induced exocytosis. Journal of Ethnopharmacology 1995;48(2):61–76. 8583796 10.1016/0378-8741(95)01285-l

[2] Oury TD Thakker K Menache M Chang LY Crapo JD Day BJ Attenuation of bleomycin-induced pulmonary fibrosis by a catalytic antioxidant metalloporphyrin. Am J Respir Cell Mol Biol 2001;25(2):164–9. 11509325 10.1165/ajrcmb.25.2.4235

[3] Vyalov SL Gabbiani G Kapanci Y Rat alveolar myofibroblasts acquire alpha-smooth muscle actin expression during bleomycin-induced pulmonary fibrosis. Am J Pathol 1993;143(6):1754–65. 7504890 PMC1887256

[4] Farber JL Rubin E editors. Essential pathology. JB Lippincott Company; 1990: pp.41–56.

[5] Brewis RAL Respiratory medicine. Vol 1 2th ed Saunder, London,1995; pp.89–91, 630–634.

[6] Reis PE Carvalho EC Bueno PC Bastos JK Clinical application of Chamomilla recutita in phlebitis: dose response curve study. Rev Lat Am Enfermagem 2011;19(1):3–10. 21412623 10.1590/s0104-11692011000100002

[7] Kato A Minoshima Y Yamamoto J Adachi I Watson AA Nash RJ Protective effects of dietary chamomile tea on diabetic complications. J Agric Food Chem 2008;56(17):8206–11. 18681440 10.1021/jf8014365

[8] Gomaa A Hashem T Mohamed M Ashry E Matricaria chamomilla extract inhibits both development of morphine dependence and expression of abstinence syndrome in rats. J Pharmacol Sci 2003;92(1):50–5. 12832855 10.1254/jphs.92.50

[9] Avallone R Zanoli P Puia G Kleinschnitz M Schreier P Baraldi M Pharmacological profile of apigenin, a flavonoid isolated from Matricaria chamomilla. Biochem Pharmacol 2000;59(11):1387–94. 10751547 10.1016/s0006-2952(00)00264-1

[10] Kuppusamy UR Das NP Effects of flavonoids on cyclic AMP phosphodiesterase and lipid mobilization in rat adipocytes. Biochem Pharmacol 1992;44(7):1307–15. 1384499 10.1016/0006-2952(92)90531-m

[11] Herfindal ET Gourley DR Textbook of therapeutics: drug and disease management. Williams & Wilkins; 1996: p. 906.

[12] Wyngarden Jb Smith L Bennett JC Cecil textbook of medicine. 19th ed W.B. Saunders company, Philadelphia, 1992:pp.1060–6

[13] Kumar V Cotran RS Robbins SL Basic pathology. 7-th edition Saunders Company Philadelphia 1997:52.

[14] Bhaskaran N Shukla S Srivastava JK Gupta S Chamomile: an anti-inflammatory agent inhibits inducible nitric oxide synthase expression by blocking RelA/p65 activity. Int J Mol Med 2010;26(6):935–40. 21042790 10.3892/ijmm_00000545 PMC2982259

[15] Srivastava JK Pandey M Gupta S Chamomile, a novel and selective COX-2 inhibitor with anti-inflammatory activity. Life Sci 2009;85(19–20):663–9. 19788894 10.1016/j.lfs.2009.09.007 PMC2784024

[16] Petroianu G Szoke E Kalász H Szegi P Laufer R Benko B Monitoring by HPLC of chamomile flavonoids exposed to rat liver microsomal metabolism. Open Med Chem J 2009;3:1–7. 19707521 10.2174/1874104500903010001 PMC2729991

[17] Srivastava JK Gupta S Antiproliferative and apoptotic effects of chamomile extract in various human cancer cells. J Agric Food Chem 2007;55(23):9470–8. 17939735 10.1021/jf071953k

[18] Gardiner P Complementary, holistic, and integrative medicine: chamomile. Pediatr Rev 2007;28(4):e16–8. 17400821 10.1542/pir.28-4-e16

[19] Srivastava JK Pandey M Gupta S Chamomile, a novel and selective COX-2 inhibitor with anti-inflammatory activity. Life Sci 2009;85(19–20):663–9. 19788894 10.1016/j.lfs.2009.09.007 PMC2784024

[20] Paulsen E Contact sensitization from Compositae-containing herbal remedies and cosmetics. Contact Dermatitis 2002;47(4):189–98. 12492516 10.1034/j.1600-0536.2002.470401.x

[21] De Smet PA Herbal remedies. N Engl J Med 2002;347(25):2046–56. 12490687 10.1056/NEJMra020398

[22] Federici E Multari G Gallo FR Palazzino G Herbal drugs: from traditional use to regulation. Ann Ist Super Sanita 2005;41(1):49–54. 16037650

[23] Rodriguez-Fragoso L Reyes-Esparza J Burchiel SW Herrera-Ruiz D Torres E Risks and benefits of commonly used herbal medicines in Mexico. Toxicol Appl Pharmacol 2008;227(1):125–35. 18037151 10.1016/j.taap.2007.10.005 PMC2322858

